# Advances in Nanotechnology for the Treatment of Herpes Virus Infections

**DOI:** 10.3390/v18030351

**Published:** 2026-03-13

**Authors:** Yohan Oliveira de Carvalho, Bruna Coelho de Almeida, Gabriela Lopes Gama e Silva, Tatielle do Nascimento, Mariana Sato de Souza Bustamante Monteiro, Eduardo Ricci-Junior

**Affiliations:** Laboratório de Desenvolvimento Galênico (LADEG) e Nanoformulações, Faculdade de Farmácia, Universidade Federal do Rio de Janeiro, Rio de Janeiro 21941-902, RJ, Brazil; yohanddl@gmail.com (Y.O.d.C.); almeidabrunacoelho@hotmail.com (B.C.d.A.); gabrielalgs95@gmail.com (G.L.G.e.S.); tatiellenascimento94@gmail.com (T.d.N.); mari-sato@hotmail.com (M.S.d.S.B.M.)

**Keywords:** nanotechnology, nanoparticles, herpes, virus, infections

## Abstract

Herpes simplex virus (HSV) infections present a major global health burden due to their high morbidity. Conventional therapies offer limited efficacy due to poor bioavailability, the need for frequent administration and potential drug resistance. Recent advances in nanotechnology provide opportunities to overcome these limitations. This review summarizes the latest advances in nanocarrier-based formulations, highlighting their role in improving bioavailability, sustained release, mucosal penetration and antiviral activity. An integrative search was conducted from January 2010 to December 2025. Inclusion and exclusion criteria were used to select the articles. After analyzing the articles, 34 were included in this review with in vitro studies and 14 with in vivo assays. These articles were evaluated in relation to physicochemical characterization studies and in vitro and in vivo assays. Studies were found involving polymeric nanoparticles, metal nanoparticles, solid lipid nanoparticles, liposomes, niosomes, nanoemulsions and nanofibers. Regarding in vitro assays, it was observed that the nanosystems showed increased antiviral activity in cell cultures infected with the herpes simplex virus. In addition, developed nanosystems showed prolonged antiviral activity and lowered toxicity in animal models. Thus, these systems prove to be effective when compared to conventional therapy and can be considered an advance in HSV infection therapy.

## 1. Introduction

Herpesviruses are a group of DNA viruses characterized by a bilayer lipid envelope and a highly conserved, complex genome. Among them, herpes simplex virus (HSV) belongs to the Alphaherpesvirinae subfamily of the Herpesviridae family and genus Simplexvirus. There are two serotypes, *Human alphaherpesvirus 1* (HSV-1) (Herpes simplex virus type 1) and *Human alphaherpesvirus* 2 (HSV-2) (Herpes simplex virus type 2). Although their genomic structures are similar, they differ significantly in transmission routes, associated diseases, and sites of latency. HSV-1 is primarily transmitted through oral contact, typically causing oral and labial herpes, but it may also be responsible for genital herpes, herpetic pharyngitis, herpetic whitlow, and herpetic encephalitis. Following infection, HSV-1 establishes latency mainly in the trigeminal ganglia, with reactivation often triggered by factors such as immunosuppression. In contrast, HSV-2 is predominantly a sexually transmitted pathogen and the leading cause of recurrent, painful, and contagious genital herpes. Beyond genital infections, HSV-2 can also result in severe complications, including neonatal herpes and meningitis. A hallmark of HSV infection is its capacity to establish lifelong latency in neuronal ganglia with periodic reactivation, which renders conventional therapies unable to eradicate the viral reservoir, posing a major challenge for effective treatment and long-term disease control [1,2,3].

HSV infection is highly widespread worldwide. According to the World Health Organization (WHO), an estimated 3.8 billion people under age 50 (around 64%) are infected with HSV-1, primarily causing oral herpes, whereas approximately 520 million people aged 15–49 (about 13%) are estimated to harbor HSV-2, the main cause of genital herpes [4,5]. Not all infections are symptomatic: in 2020, about 205 million individuals (5.3%) experienced at least one symptomatic episode of genital herpes, of which an overwhelming majority (92%) were attributed to HSV-2. These figures underscore both the silent and visible burden of HSV infections and highlight the significant challenge they pose to public health [4,5].

Nanotechnology, through the development of nanoscale drug delivery systems, offers an innovative solution to the challenges associated with traditional HSV therapies. By enabling targeted, sustained, and efficient drug delivery, nanotechnology can enhance the therapeutic outcomes of antiviral treatments while reducing administration frequency and minimizing adverse effects [3,6,7,8,9,10]. In addition, nanocarriers can encapsulate antiviral drugs, protecting them from enzymatic degradation, enhancing mucosal adhesion and penetration and, consequently, improving their bioavailability [3,10,11,12,13]. Nanotechnology enables the co-administration of multiple agents (antivirals, anti-inflammatories or immunomodulators) and after topical administration the drugs can be released, acting on different mechanisms to combat the virus [3,6,7,9,10,14].

The most commonly used antiviral nanocarriers for the treatment of herpes are liposomes [15,16,17,18], nanoparticles [19,20,21] and nanofibers [22,23]. Furthermore, metal nanoparticles, particularly silver nanoparticles (AgNPs) and gold nanoparticles (AuNPs), have demonstrated broad-spectrum antiviral activity through multiple mechanisms, including inhibition of viral entry, interference with replication, and disruption of viral assembly [24,25]. AgNPs can bind to viral envelope glycoproteins, preventing attachment and penetration into host cells, while also generating reactive oxygen species that damage viral components.

Similarly, AuNPs have been shown to block viral adsorption and fusion, and when functionalized with specific ligands, they enhance targeted antiviral effects by inhibiting genome replication and protein synthesis. ZnO-based nanoparticles (ZnONPs) can act as an efficient anti-HSV agent by preventing viral infection [26]. Collectively, these findings highlight metal nanoparticles as promising nanoplatforms for developing novel antiviral therapies [24,25,26].

Carbon materials such as carbon quantum dots (CQDs) are able to interact with surface glycoproteins, preventing viral attachment to host cells [27,28].

This review aims to highlight recent advances and limitations in the treatment of herpes infections through nanotechnology. It discusses diverse strategies for drug delivery using nanocarriers, including the incorporation of photosensitizers to enhance antiviral photodynamic therapy. By focusing on innovative nanoformulation approaches, this review highlights the potential of nanotechnology to combat the herpes virus, aiming to expand treatment options against this viral infection.

## 2. Methods

### 2.1. Focused Question

The primary research questions guiding this literature review are as follows:

(1) Is nanotechnology effective in enhancing the treatment of Herpes virus infections?
(2) Are antivirals encapsulated in nanocarriers more efficient than free drug?
(3) What are the recent advances in in vitro studies involving nanocarriers loaded with antivirals?
(4) What is the antiviral mechanism of action of inorganic nanoparticles, dendrimers and carbon-based nanoparticles?
(5) Is there translational research with antivirals encapsulated in nanocarriers for use in the treatment of Herpes?

### 2.2. Search Strategy

An integrative search was conducted in the Scopus, Web of Science, and PubMed databases (January 2010 to December 2025). The following keywords and Boolean operator combinations were applied: “Nanotechnology” AND “Herpes”. PubMed, Scopus and Web of Science differ in their disciplinary coverage, access, analytical tools and indexing depth, with PubMed specializing in biomedical and medical literature, offering free access. Scopus and Web of Science are multidisciplinary databases providing advanced citation analysis and metrics. The Science Direct database was not used, as the authors chose to use Scopus, both Elsevier platforms, thus avoiding the existence of many duplicate references. The active search in the databases and the application of the inclusion/exclusion criteria were performed in triplicate (*n* = 3 determinations) by the co-authors of this review article to validate the number of articles found and eligible.

### 2.3. Eligibility Criteria

The study selection process followed strict inclusion and exclusion criteria to ensure relevance and quality. Inclusion criteria encompassed (i) experimental studies, whether in vitro or in vivo, and (ii) direct comparisons of antiviral efficacy between free drugs and drugs encapsulated in nanocarriers. Exclusion criteria were applied to eliminate (i) duplicate publications, (ii) secondary literature (review articles, conference abstracts, books, book chapters, patents) and (iii) studies unrelated to nanotechnology or Herpes. After the initial Boolean search, a two-step screening was conducted: first, articles were assessed for relevance based on titles and abstracts; subsequently, full texts were reviewed to confirm alignment with the research objectives. This approach ensured a focused analysis of high-impact, directly applicable studies [29,30,31,32].

## 3. Results and Discussion

### 3.1. Search Strategy

**Figure 1:**
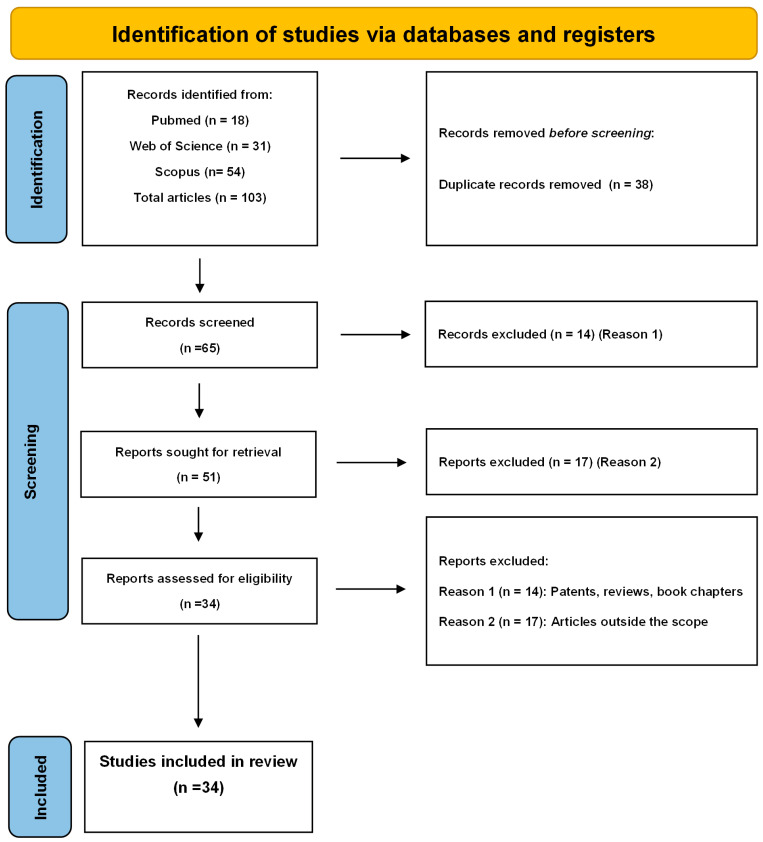
Flowchart developed to carry out the literature search. The flowchart shows the methodology used to select the articles for this review [33].

The search using the keywords mentioned above yielded a total of 103 articles in the databases used, 54 of which came from Scopus, 31 from Web of Science and 18 from PubMed. Of this total, 38 duplicate articles were found, reducing the total number to 65 articles. The first mapping to exclude articles found that, out of the 65 remaining articles, 31 had to be eliminated because they were part of the exclusion criteria: Reason 1 (patents, review articles, abstracts for scientific events), and Reason 2 (articles outside the scope), leaving 34 articles eligible for this review. Figure 1 shows the flowchart developed to carry out the search methodology. Only articles that presented systems on a nanometric scale and with tests on the Herpes simplex virus species were selected. This analysis resulted in a list of 34 articles that were evaluated concerning the type of nanocarriers used, their composition, the drug and concentration used in the treatment, the method of preparation, as well as the methods of physicochemical characterization of the nanosystems (Table 1).

The selected articles were also evaluated in relation to in vitro assays. The viral type used (HSV-1 and/or HSV-2), the assays performed to evaluate antiviral activity, and the results obtained were analyzed. These results can be seen in Table 1. Out of the 34 articles analyzed, only 14 presented in vivo tests, and these are listed in Table 2, which gives an overview of the in vivo experiments presented.

### 3.2. Antiviral Mechanism of Nanomaterials

The antiviral activity of nanocarriers can be mediated through three main mechanisms: (1) direct viral inactivation via physical interaction between the nanomaterial and the viral particle, as reported for nanosystems based on dendrimers, metallic nanoparticles (AgNPs, AuNPs, ZnO), and carbon-based nanomaterials such as fullerenes and carbon nanodots; (2) antibody-mediated viral neutralization, in which immunonanoparticles functionalized with anti-HSV monoclonal antibodies bind specifically to the viral surface, thereby preventing viral adsorption and entry into host cells; and (3) nanocarriers for drug delivery, including synthetic or natural antivirals as well as biomolecules for gene therapy, such as small interfering RNA (siRNA), enabling intracellular inhibition of viral replication [3,6,7,9,10,11,12,13].

Dendrimers are highly branched macromolecules that can be functionalized with sulfonated groups capable of binding to the viral surface, irreversibly inactivating HSV viruses. Furthermore, dendrimers are able to block the adsorption, entry, and fusion of the viral particle to the host cell [3].

Metallic nanoparticles are an interesting alternative in the fight against the herpes virus [24,25]. Metallic nanoparticles exhibit interesting bionanotechnological properties such as small size, large surface area, reactivity that allows modification of the nanosystem surface with chemical groups or antiviral drugs, and finally, biocompatibility. AgNPs can physically interact with free viral particles or with viruses already bound to the host cell. Through these interactions, AgNPs may exert a direct virucidal effect, leading to inactivation of the infectious particle or inducing morphological alterations of the virion. In addition, AgNPs can interfere with the early stages of viral replication, including viral attachment to the host cell and subsequent penetration processes [64]. The antiviral efficacy of AgNPs was observed in studies developed by Fayaz et al. (2012), Gaikwad et al. (2013), Kryzowska et al. (2023), Pan et al. (2022) and Kryzowska et al. (2022) [45,46,47,48,51].

ZnO NPs with potential anti-HSV activity were also produced [52,53]. ZnO NPs showed virucide action by stimulating the formation of reactive oxygen species and the release of zinc ions that are toxic to the virus, as well as interacting with and causing damage to the viral structure [26].

AuNPs possess anti-HSV activity, primarily blocking viral entry and exerting virucidal effects, with excellent biocompatibility. The surface properties are interesting because they can be modified with antiviral chemical groups, immunomodulators, and drugs such as acyclovir. AuNPs exhibit potent anti-HSV activity, mainly through inhibition of viral entry and direct virucidal effects, while maintaining excellent biocompatibility. Their surface chemistry is highly versatile, allowing functionalization with antiviral groups, immunomodulators, or conventional drugs such as acyclovir to enhance therapeutic efficacy. Mechanistically, AuNPs can interact with HSV envelope glycoproteins (gB, gC, and gD), thereby preventing their binding to host–cell heparan sulfate proteoglycan receptors. This interference disrupts virus–cell receptor interactions and effectively inhibits viral adsorption, membrane fusion, and entry into host cells. Consequently, AuNP-based systems demonstrate pronounced antiviral effects, particularly in pretreatment and co-treatment strategies [3,24,25,26].

Fullerenes (C60) are carbon-derived materials with unique surface properties that enable functionalization with hydroxylated, carboxylated, or cationic chemical groups exhibiting antimicrobial and antiviral activity. Thus, fullerenes are capable of blocking virus–cell receptor interactions through direct binding to viral glycoprotein-based receptors, thereby preventing adsorption, fusion, and entry of the viral particle into the host cell. The antiviral mechanism of fullerenes is primarily observed in virus pre-treatment assays prior to contact with the host cell [3,27,28].

Carbon dots (CDs) are carbon-based nanomaterials and exhibit antiviral action. Carbon dots (CDs) exert antiviral activity primarily through direct binding to viral surface components, which effectively block viral particles and prevent their interaction with host cell surface molecules. This interaction disrupts critical early stages of the viral life cycle, including adsorption, membrane fusion, and subsequent entry into the host cell. The antiviral mechanisms involving: (1) viral inactivation by directly binding of the CDs to viral surface proteins (glycoproteins) through electrostatic interaction; (2) viral inactivation by surface functionalization with chemical groups based in amine, carboxyl, boronic acid, or sulfonate groups, which mimic host–cell receptors and promote the binding of CDs to the viral surface; and (3) drug delivery by surface functionalization of the CDs with antiviral actives, siRNA for gene therapy, and immunomodulators [3,27,28].

Encapsulation of antiviral agents in nanocarrier systems, like polymeric nanoparticles, solid lipid nanoparticles, liposome, niosome, nanoemulsions, nanogels, nanofibers and nanowires, represent a promising strategy for the topical treatment of herpes infections [3,15,16,17,18,19,20,21,22,23]. Acyclovir, one of the most widely used anti-herpetic drugs, is limited by poor aqueous solubility and low skin permeation, which substantially restricts its topical bioavailability [3]. In addition, systemic administration of acyclovir and its prodrug valacyclovir has been associated with adverse effects, including neurological and renal complications. Nanocarrier-based delivery systems can enhance drug permeation through the skin, thereby increasing local bioavailability at the site of infection. Moreover, these systems enable sustained drug release, which may reduce dosing frequency and minimize systemic exposure and associated side effects. There are several studies that encapsulate drugs in nanocarriers in order to improve cutaneous bioavailability and antiviral action [14,34,58,59,60,61].

siRNA can silence genes with excellent specificity promoting gene therapy. However, free siRNA is rapidly degraded by enzymes in blood and tissues becoming biologically inactive. This biomolecule requires the protection of a nanocarrier to reach the target site. Nanoparticles provide protection, transport, cellular entry, and cytoplasmic release, making siRNA therapeutically viable [16,17,36]. The use of siRNA was also observed in the studies of Jbara-Agbaria et al. (2022) and Steinbach et al. (2012) for viral inactivation of herpes virus [16,36].

### 3.3. Nanocarriers

Nanocarriers are a nanoscale delivery system designed to transport active pharmaceutical ingredients, such as drugs, proteins, genes or vaccines. Its main purpose is to encapsulate, protect and direct the therapeutic agent to the target site within the body, thereby enhancing bioavailability, controlling drug release, reducing systemic side effects and improving therapeutic efficacy. Characteristics such as nanometric size provide a larger surface area and enhanced physicochemical properties not observed at the macroscopic level, enabling more efficient interactions with biological cells and tissues [3,65,66].

There is a wide range of nanosystems in our study, including polymeric nanoparticles [14,34,35,36,37,38,39,40,41,42,43,44], inorganic/metallic/composite nanoparticles [28,45,46,47,48,49,50,51,52,53], fullerene/carbon nanodots [54,55,56] liposomes/niosomes [16,17,18,57,58], nanoemulsions [59,60,61] and nanofibers/nanowires [62,63], which are generally composed of biocompatible and biodegradable materials such as poly(lactic-co-glycolic acid) (PLGA), chitosan, lipids and surfactants. These systems represent one of the major innovations in pharmaceutical nanotechnology, significantly contributing to the development of safer, more effective, and more targeted therapeutic strategies.

**Figure 2:**
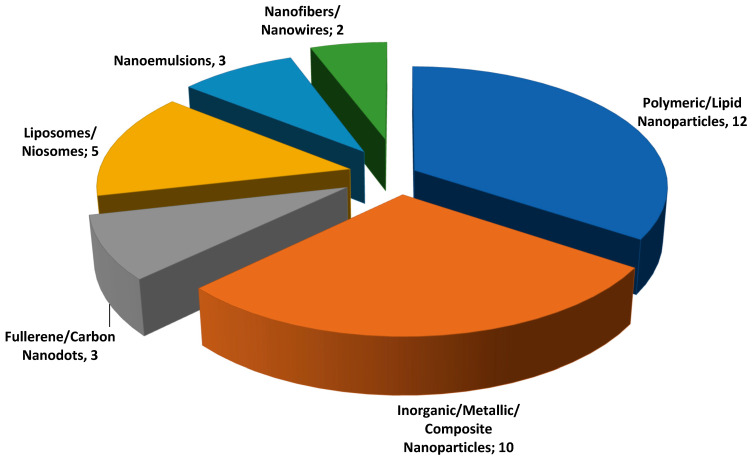
Types of nanomaterials used in the 35 selected articles and their percentage in relation to the total.

Figure 2 shows the types of nanocarriers developed for the treatment of HSV. Based on the analysis of the selected articles, majority of the nanocarriers developed were polymeric/lipid nanoparticles (12) and inorganic/metallic/composite nanoparticles (10), followed by liposomes/niosomes (5), fullerene/carbon nanodots (3), nanoemulsions (3), and nanofibers/nanowires (2).

In the current context, nanotechnology has emerged as a promising strategy against HSV, mainly due to its ability to encapsulate antiviral agents, providing greater control over drug release and bioavailability. Based on the reviewed literature, among the various types of nanocarriers, polymeric nanoparticles stand out for their versatility, tunable physicochemical properties and ease of surface modification. These nanoparticles are colloidal systems with dimensions ranging from 1 to 1000 nm, generally composed of natural, synthetic, or semi-synthetic polymers such as PLGA, chitosan and polyethylene glycol (PEG), and may also include substances derived from green chemistry [64,65,66,67].

In addition to polymeric nanoparticles, liposomes and niosomes also exhibit excellent potential as drug delivery systems, as they provide amphiphilic environments suitable for the efficient encapsulation of antiviral agents while maintaining high biocompatibility. Liposomes are spherical vesicles composed of one or more phospholipid bilayers surrounding an aqueous core. This amphiphilic structure enables the encapsulation of hydrophilic drugs (in the aqueous core) and lipophilic drugs (in the phospholipid bilayer), being widely used due to their high biocompatibility and low toxicity [68,69]. Owing to their bilayer structure, liposomes are notable not only for transporting antiviral agents but also for carrying molecules such as siRNA, viral particles, total carrageenan, a mixture of κ-, ι- and λ-carrageenans (κ-CRG/Ech and Σ-CRG/Ech) complexes [18] and genes related to Varicella zoster virus (VZV ORF7) [17]. Thus, they combine therapeutic efficacy with safety, making them promising candidates for advanced antiviral applications [16,17,18,57,58].

Niosomes, in turn, are vesicles composed of lipid bilayers formed by nonionic surfactants and cholesterol, displaying biodegradable and biocompatible characteristics. They are capable of encapsulating both hydrophilic and hydrophobic compounds, protecting the active ingredient, increasing solubility, reducing toxicity and promoting controlled and prolonged drug release [69]. Due to these properties, niosomes have emerged as a promising alternative to liposomes, offering greater physical stability and lower production costs [58,69].

Other nanostructures, such as nanoemulsions and nanofibers, have also shown promising results in the treatment of HSV infections. Nanoemulsions and nanostructured hydrogels have been extensively studied for their ability to enhance the solubility and bioavailability of poorly water-soluble antiviral agents. Nanoemulsions are colloidal systems consisting of two immiscible phases (oil and water) stabilized by surfactants, which provide greater stability, transparency and absorption of bioactive compounds [59,60,61,70]. Nanofibers, in contrast, are one-dimensional solid structures with diameters generally below 1000 nm, featuring a high surface area-to-volume ratio and excellent porosity. These characteristics confer high drug-loading capacity and controlled-release behavior, making them particularly suitable for topical and localized therapeutic applications [62,63,71,72,73]. In the reviewed studies, several components have been used in the formulation of these nanoemulsions and nanofibers, including chitosan, clove oil, castor oil, Tween 80, Span 80, propylene glycol, polyethyleneglycol-6 oleate (Myo-6V) and organosiloxane, all of which contribute to the stability, permeability and antiviral efficacy of the formulations [59,60,61,62,63].

In summary, although polymeric nanoparticles remain the most versatile and widely studied nanocarriers for the treatment of HSV, other platforms, such as liposomes, niosomes, nanoemulsions and nanofibers, offer complementary approaches adaptable to different administration routes and therapeutic purposes. Collectively, these nanostructures provide enhanced stability, controlled release, improved bioavailability and great antiviral efficacy, representing a significant advancement in next-generation therapies for Herpes simplex virus infections.

### 3.4. Preparation Methods and Characterization

The articles selected for this review developed various nanocarrier systems aimed at improving therapy against HSV. Among these systems are nanoparticles, liposomes, niosomes, nanoemulsions and nanofibers. The preparation techniques varied depending on the type of nanocarrier, the presence or absence of an active pharmaceutical ingredient and the components employed.

Among the nanoparticles studied, polymeric, metallic, solid lipid, and polymeric micelle-based systems were the most prominent [14,34,35,36,37,38,39,40,41,42,43,44]. The main preparation techniques reported include high-pressure homogenization, green synthesis, emulsification and solvent evaporation, hot high-shear homogenization and ultrasonication, nanoprecipitation, and bath sonication. The high-pressure homogenization technique consists of a mechanical process in which fluids are homogenized in a single step while being subjected to high pressure. This method generates intense shear forces, allowing particle size reduction and the formation of more stable emulsions [74,75].

In green synthesis, natural and eco-friendly materials, such as microorganisms or plant extracts, are used in the preparation of nanomaterials. In the presence of these biological agents, metallic nanoparticles can be developed. This technique is characterized as non-toxic, environmentally friendly, cost-effective and more sustainable. However, it also presents challenges, including the extraction of raw materials, long synthesis times and the generally non-uniform size distribution of the resulting nanoparticles [76,77,78].

The emulsion solvent evaporation technique is widely employed for the encapsulation of lipophilic drugs into polymeric nanoparticles. Typically, the lipophilic drug and polymer are solubilized in an organic solvent, followed by the formation of an emulsion with an aqueous surfactant solution. Subsequently, the solvent is evaporated, leading to polymer precipitation and the formation of polymeric nanoparticles that encapsulate the drug. In this method, high-pressure homogenizers and ultrasonic homogenizers may be used during the emulsification step. Ultrasonic homogenizers (or sonicators) employ ultrasound waves and shear forces to generate alternating low- and high-pressure cycles, resulting in the disruption of larger droplets into smaller ones and promoting stable emulsion formation. The hot high-shear homogenization technique involves heating a lipid phase followed by the addition of a lipophilic compound. An aqueous phase containing a surfactant is heated to the same temperature, and both phases are then mixed and processed at high speed to form nanoparticles [79,80,81,82,83].

In the nanoprecipitation technique, the polymer and the lipophilic active compound are solubilized in an organic solvent that is miscible with water. This organic solution is then added to an aqueous solvent under constant stirring. As the organic solvent diffuses into the aqueous phase, nanoparticle precipitation occurs [84,85,86].

Liposomes and niosomes were developed using the lipid film hydration technique [16,18,57,58]. This method is widely employed in the preparation of vesicular systems such as liposomes and niosomes due to its simplicity and reproducibility. Nanoemulsions, on the other hand, were prepared using emulsification assisted by sonication [59,60,61], while nanofibers were produced by the electrospinning technique [62,63].

Various techniques can be employed for the characterization of nanosystems. The main analytical methods reported in the reviewed studies include particle size and polydispersity index analysis, surface charge determination (zeta potential), morphological analysis, encapsulation efficiency determination and in vitro drug release profile studies. These analyses are essential for evaluating the effectiveness of nanosystem formation, assessing homogeneity and stability, and predicting their potential behavior in vivo after administration.

The particle size and polydispersity index (PDI) are directly related to the stability of the developed formulations, as well as to their pharmacokinetic and pharmacodynamic parameters in vivo. It is well known that nanosystems in the nanometric range exhibit different biological behaviors depending on their size. Nanosystems smaller than 20 nm tend to be more easily eliminated via the renal route, whereas those larger than 200 nm are often recognized and cleared by the phagocytic system [87,88,89]. Regarding the PDI, higher values indicate a more polydisperse and heterogeneous formulation, which negatively affects its stability. PDI values below 0.3 are typically considered indicative of homogeneous and monodisperse systems [90,91,92].

Another critical parameter influencing nanosystem stability is the surface charge, expressed as zeta potential. Nanocarriers with high surface charge values, either positive or negative, tend to exhibit greater stability due to the electrostatic repulsion between suspended particles, which reduces the likelihood of aggregation and coalescence. This parameter is influenced by the composition of the nanosystem, such as the use of charged polymers in the preparation of polymeric nanoparticles or the inclusion of surface-modifying agents to alter nanocarrier properties [93,94].

Regarding the studies reviewed that focused on nanoparticle development, most reported the production of polymeric and metallic nanoparticles. Among the polymeric nanoparticles, formulations based on PLGA, PLA and chitosan were the most common, as these are biodegradable polymers widely employed in nanocarrier design. In the case of metallic nanoparticles, studies primarily investigated gold and silver nanoparticles, which have gained attention due to their antiviral, antimicrobial and biocompatible properties.

Donalisio et al. (2019) [14] developed chitosan-stabilized nanodroplets decorated with cyclodextrin (SBEβCD) to improve stability. All formulations showed a particle size of around 400 nm, a low PDI (approximately 0.2) and a positive surface charge (between 20 mV and 33 mV) due to the cationic groups of chitosan. A decrease in zeta potential was observed for formulations modified with SBEβCD (from 32.10 ± 3.25 mV to 20.55 ± 2.44 mV for the drug-free formulations, and from 30.46 ± 3.01 mV to 21.12 ± 2.87 mV for the formulations containing valacyclovir), confirming the interaction between chitosan and SBEβCD. The encapsulation efficiency analysis showed that the system was effective in drug loading (90.5% for nanodroplets without SBEβCD and 91.2% for those containing SBEβCD). In vitro release studies showed that SBEβCD-modified nanodroplets exhibited a more sustained and slower release of valacyclovir, reaching 22% after 24 h [14].

Sangboonruang et al. (2022) [40] used PLGA nanoparticles to encapsulate propolis extract. In addition to PLGA, chitosan was used to enhance repulsion between nanoparticles and prevent aggregation. The nanoparticles presented sizes of 450 nm and 650 nm and PDI of 0.21 and 0.34 for the propolis-loaded and blank nanoparticles, respectively. The zeta potential ranged between 36 mV and 38 mV. The encapsulation efficiency of the propolis extract reached 80%. Scanning electron microscopy showed spherical-shaped nanoparticles. The in vitro release assay revealed a sustained release profile over 24 h, with approximately 30% of the propolis released from the nanoparticles [40].

Mariotti et al. (2024) [38] produced PLGA nanoparticles functionalized with an anti-HSV-2 monoclonal antibody. Particle size and zeta potential analyses by DLS confirmed an increase in particle size after antibody conjugation (from 86.6 ± 10.9 nm and −0.7 ± 0.3 mV to 151 ± 10.4 nm and −5.1 ± 1.9 mV). The polydispersity index remained below 0.2, confirming the formation of monodisperse systems [38].

Lima et al. (2018) [41] developed PLA nanoparticles for the encapsulation of chloroquine diphosphate. The nanoparticles obtained by the nanoprecipitation method exhibited a size below 250 nm, negative zeta potential, and a polydispersity index lower than 0.2. The formulations were prepared using different drug/polymer ratios (1:5, 1:10, and 1:15) at pH 6.4. It was observed that increasing the amount of polymer led to an increase in nanoparticle size (173.5 ± 8.5 nm, 189.1 ± 6.5 nm, and 226.4 ± 9.2 nm). The formulations developed at a 1:10 ratio were also reproduced at pH 11 and pH 8.4, yielding particle sizes of 200.6 ± 11.4 nm and 231.4 ± 11.5 nm, respectively. The formulations developed under acidic pH showed low encapsulation efficiency (10.6 ± 1.3%, 8.4 ± 2.6%, and 3.4 ± 1.4%), which decreased with increasing polymer concentration. When the pH of the solution was raised to 11 with 0.1 M NaOH, the encapsulation efficiency increased to 11.4 ± 2.0%. When the pH was adjusted to 8.4 with 0.5 M NaHCO_3_, the encapsulation efficiency increased to 25.0 ± 1.6% [41].

Chloroquine is a low-molecular-weight drug whose solubility is influenced by pH. At pH 6.4, the hydrophilic compound does not interact with PLA, a hydrophobic polyester. Thus, increasing the amount of polymer does not favor higher drug encapsulation. Adjusting the pH to the alkaline range can increase the non-ionized fraction of chloroquine and improve encapsulation efficiency. Alkalinizing the aqueous phase with NaOH increased encapsulation efficiency from 8.4 ± 2.6% to 11.4 ± 2.0%, while using NaHCO_3_ raised it to 25.0 ± 1.6%. This occurs because chloroquine tends to hydrolyze and degrade in 0.1 M NaOH solutions. The best formulation was obtained at a 1:10 ratio with an aqueous solution at pH 8.4. These conditions were then used to prepare new nanoparticles via the emulsion–solvent evaporation method. The resulting nanoparticles exhibited particle sizes of 283.9 ± 53.2 nm and 297.3 ± 26.1 nm, negative zeta potential (−25.4 ± 11.6 mV and −20.0 ± 12.0 mV), a PDI around 0.3 and encapsulation efficiency of 64.1 ± 5.0%. This formulation was selected for subsequent assays. Atomic force microscopy showed spherical morphology, and the in vitro release assay revealed a slow and sustained release profile over 10 h [41].

Among other polymeric nanoparticles developed, Ensign et al. (2014) [34] prepared polystyrene nanoparticles. The commercially obtained nanoparticles exhibited particle sizes of 193 ± 3 nm and 485 ± 1 nm and surface charges of −55.0 ± 2.8 mV and −57.7 ± 1.1 mV. These nanoparticles were modified with PEG, which resulted in a slight increase in size as expected due to surface modification. The PEGylated nanoparticles exhibited particle sizes of 232 ± 4 nm and 500 ± 2 nm, with an increase in surface charge to −1.9 ± 0.2 mV and −8.0 ± 0.4 mV. Additionally, PLGA nanoparticles were produced, showing particle sizes of 112 ± 1 nm and a zeta potential of −5.9 ± 0.8 mV [34].

These data, along with other examples of polymeric nanoparticles developed, are summarized in Table 1.

Krzyzowska et al. (2022) [51] developed silver and gold nanoparticles functionalized with lactoferrin. Particle size analyses showed the formation of silver nanoparticles of 11 ± 3 nm (10 nm AgNPs) and 37 ± 12 nm (30 nm AgNPs), and gold nanoparticles of 12 ± 4 nm (10 nm AuNPs). The silver nanoparticles exhibited zeta potentials of −48.0 mV (10 nm AgNPs) and −53.3 mV (30 nm AgNPs), while gold nanoparticles showed −54.0 mV (10 nm AuNPs). Two different methodologies were used for silver nanoparticle synthesis, resulting in formulations with distinct particle sizes and zeta potentials. The lactoferrin-functionalized silver nanoparticles presented particle sizes of 23 ± 9 nm and 45 ± 18 nm and zeta potentials of −35.6 mV and −25.4 mV. The functionalized gold nanoparticles displayed two peaks in size distribution (174 ± 85 nm and 977 ± 533 nm), indicating the formation of aggregates. Scanning electron microscopy revealed spherical morphology [51].

Krzyzowska et al. (2023) [47] also developed silver nanoparticles modified with epigallocatechin gallate (EGCG) and tannic acid (TA). Epigallocatechin gallate is a flavonoid with antiviral activity that inhibits viral binding and cell entry by interacting with membrane proteins of both the virus and host cell, and it also interferes with viral replication. Tannic acid can inhibit viral attachment, entry, and spread to other cells. The nanoparticles were characterized by scanning electron microscopy, revealing spherical morphology. Particle sizes were 30 ± 10 nm (AgNPs), 32 ± 11 nm (EGCG-AgNPs), and 35 ± 10 nm (TA-AgNPs). Zeta potential results were −76.0 mV (AgNPs), −67.0 mV (EGCG-AgNPs), and −58.0 mV (TA-AgNPs) [47].

Halder et al. (2018) [49] synthesized gold nanoparticles stabilized with gallic acid. Gallic acid acted as an antioxidant agent that interacted favorably with the nanoparticles, enhancing their stability. These nanoparticles exhibited an average particle size of 18.27 nm and a polydispersity index of 0.148. The zeta potential analysis revealed a surface charge of −21.7 mV. Transmission electron microscopy showed uniform and spherical particles [49].

These data, along with other examples of metallic nanoparticles developed, are also presented in Table 1.

In addition to polymeric and metallic nanoparticles, other classes of nanoparticles were also reported in the reviewed studies, including inorganic nanoparticles (such as tin oxide, zinc oxide and selenium nanoparticles), plant-derived nanoparticles and solid lipid nanoparticles. These data are summarized in Table 1.

Studies developed by Klimova et al. (2020) [54], Fedorova et al. (2012) [55] and Barras et al. (2016) [56] presented fullerene-based carbon nanoparticles and carbon nanodots for evaluating antiviral activity. Nanosystems below 100 nm were obtained, and with a low polydispersity index (<0.3) [54,55,56].

Regarding liposome and niosome-based systems, 5 studies were identified that developed these vesicular nanocarriers (Table 1).

Jbara-Agbaria et al. (2022) [16] developed liposomes for the encapsulation of siHSV, a siRNA that inhibits the gene expression of the infected cell protein 0 (ICP0), an important regulatory protein in viral infection. ICP0 promotes the transcription of viral genes and assists in the evasion of host antiviral defenses. Various liposomal formulations were prepared by varying the preparation method, lipid composition, and the ratio between cationic lipids and siHSV. Particle sizes ranged from 107 ± 5.0 nm to 194 ± 2.6 nm; PDI values ranged from 0.11 ± 0.01 to 0.25 ± 0.03; zeta potentials varied from −7.4 ± 0.6 mV to 4.7 ± 0.3 mV and encapsulation efficiency values ranged from 25 ± 5% to 89 ± 7%. The optimal formulations were obtained with lipid-siHSV ratios of 16:1 and 5:1, showing particle sizes of 142 ± 1 nm and 133 ± 2 nm, respectively. The corresponding PDI values were 0.21 ± 0.03 and 0.19 ± 0.02, zeta potentials were 2.7 ± 0.1 mV and 3.3 ± 0.2 mV, and encapsulation efficiencies were 86 ± 10% and 89 ± 7%, respectively. Microscopic images revealed uniform and spherical vesicles. Since the 5:1 ratio liposomes exhibited higher encapsulation efficiency, they were selected for further studies [16].

Parsa et al. (2014) [58] developed niosomes for acyclovir encapsulation. Particle sizes ranged from 122.6 ± 0.2 nm to 987 ± 0.7 nm depending on the proportions of Span 20, Span 60, and cholesterol. Niosomes prepared with Span 60 exhibited smaller particle sizes. It is known that vesicle size decreases with the increasing hydrophobicity of surfactants, which depends on their hydrophilic–lipophilic balance (HLB); Span 20 has an HLB of 8.6, while Span 60 has an HLB of 4.7, explaining these results. Furthermore, the addition of negatively charged molecules (dicetyl phosphate) contributed to preventing aggregation through electrostatic repulsion and to reducing niosome size. Additionally, niosomes were formulated with α-tocopheryl polyethylene glycol 1000 succinate (TPGS) to improve acyclovir encapsulation efficiency. TPGS is an amphiphilic form of vitamin E capable of emulsifying hydrophobic compounds. Consequently, the niosomes prepared with TPGS exhibited the highest encapsulation efficiency. The in vitro release assays revealed a slow and sustained drug release profile for the TPGS-based niosomes, which may have resulted from the interaction between acyclovir and TPGS that delayed the drug release process [58].

Studies involving nanoemulsions were also identified. Al-Subaie et al. (2015) [60] developed acyclovir-loaded nanoemulsions in hydrogel form to enhance drug absorption. Several formulations were produced by varying oil and surfactant concentrations. The resulting droplet sizes ranged from 41 nm to 241 nm [60].

Regarding nanofibers, Szymańska et al. (2022) [62] developed a chitosan–poly(ethylene oxide) nanofibrous mat as a vaginal delivery platform for tenofovir. Both drug-free and drug-loaded nanofibers exhibited uniform white surfaces with thicknesses ranging from 100 μm to 200 μm. Scanning electron microscopy revealed three-dimensional, randomly oriented, entangled structures with predominantly smooth surfaces. The preparation method was effective in producing nanofibers with an encapsulation efficiency of approximately 70% [62].

### 3.5. Drugs and Other Active Ingredients

The analyzed literature, encompassing 35 studies, highlights a wide variety of nanocarriers developed to optimize the delivery of antiviral agents against HSV. These systems have been employed to deliver a range of drugs and bioactive substances, including acyclovir, valacyclovir, dimethyl fumarate, ethanolic propolis extract, essential oils, metallic particles such as silver and gold, inorganic compounds such as zinc oxide and selenium, and biomolecules like siRNA targeting HSV genes. Collectively, these nanocarriers demonstrate strong potential to enhance bioavailability, promote controlled release and antiviral efficacy across various experimental models.

Among the most extensively investigated antivirals are acyclovir (ACV) and valacyclovir (VACV) [14,42,44,58,59,60,61]. The use of these classic drugs in nanostructured systems aims to overcome pharmacokinetic limitations. This is the case with ACV, where its therapeutic potential is limited by low oral bioavailability due to low aqueous solubility, low permeability and short plasma half-life [95]. Similarly, VAVC, a prodrug for ACV, although developed to increase ACV absorption, undergoes rapid conversion to acyclovir via first-pass intestinal and hepatic esterases, and therefore still depends on this biotransformation for its antiviral activity. Furthermore, increasing systemic levels of acyclovir does not completely overcome limitations such as restricted tissue penetration and the inability to eradicate latent viral reservoirs [96].

In turn, tenofovir faces limitations associated with renal and bone toxicity, variable intracellular activation and low permeability across biological membranes, factors that restrict its long-term therapeutic performance [97,98]. Incorporating these agents into nanostructured delivery systems can improve their bioavailability, stability and targeting, while reducing systemic toxicity [96,97,98].

In addition to synthetic antivirals, natural compounds and bioactive substances have been extensively explored as therapeutic alternatives or adjuvant agents. Among them, dimethyl fumarate (derived from *Rheum tanguticum*), ethanolic extracts of propolis and EGCG stand out, exhibiting potent antiviral activity when incorporated into polymeric, metallic or hybrid nanoparticles [40,43,47]. Silver nanoparticles functionalized with EGCG or lactoferrin showed pronounced virucidal effects and high biocompatibility, highlighting the synergistic potential between natural compounds and inorganic platforms [47,51].

Another group of systems explored includes metallic and inorganic oxide nanoparticles, such as silver, gold, zinc oxide, and selenium, synthesized through chemical or “green” routes. These nanocarriers exhibited broad-spectrum antiviral activity, with emphasis on silver nanoparticles applied in AgNPs-coated condoms and tetrapodal zinc oxide (ZnO) nanoparticles, which demonstrated not only direct antiviral activity but also immunoprotective effects on the vaginal mucosa [45,53].

Therapeutic biomolecules, particularly siRNA, have also stood out as promising antiviral agents. The encapsulation of siRNA in cationic chitosan or PLGA nanoparticles enabled efficient in vivo gene silencing, resulting in a significant reduction in viral replication in HSV-2 infection models [36,38].

Other studies report the development of liposomes containing κ-CRG/Σ-CRG carrageenan complexes with echinochrome A (Ech) [18] or 2-aminomethyl-3-hydroxy-1,4-naphthoquinone derivatives with different substituents, which exhibited expressive antiviral activity [57]. Likewise, niosomes composed of nonionic surfactants, cholesterol, and TPGS were used to deliver acyclovir, showing controlled release and greater stability under physiological conditions [58].

Nanoemulsions, prepared by spontaneous emulsification, have also been widely employed to optimize acyclovir delivery [59,60,61]. Formulations containing vegetable oils (clove and castor), nonionic surfactants (Tween 80 and Span 80) and co-solvents such as propylene glycol and Myo-6V resulted in stable nanometric particles with enhanced solubility and cutaneous permeation of the drug [60].

Finally, nanofibers obtained by electrospinning of chitosan and poly(ethylene oxide) have been applied for local and controlled antiviral release, such as tenofovir disoproxil fumarate, exhibiting mucoadhesive properties, biodegradability, and prolonged efficacy against HSV-2 [62]. Similarly, ultrathin organosiloxane membranes and nanofibers composed of chitosan and poly(ethylene oxide) loaded with tenofovir disoproxil fumarate demonstrated precise release rate control and sustained efficacy in inhibiting HSV-2 replication in genital models [62,63].

These studies demonstrate that the incorporation of conventional drugs, natural compounds and biomolecules into various nanocarriers enhances bioavailability, release control and antiviral efficacy, which represents a significant advancement in the development of safe, effective and biocompatible therapies for the treatment of HSV infections.

### 3.6. In Vitro Studies

In vitro assays are performed to verify nanosystems efficacy, as well as to evaluate potential cytotoxicity in healthy cells. In the selected studies, the antiviral efficacy of the nanocarriers was analyzed in cells infected with HSV.

In cytotoxicity tests, healthy cells are exposed to developed formulations for a specific period, followed by an evaluation of cell viability. Among the techniques used, those employing dyes such as [3-(4,5-dimethylthiazol2-yl)-5-(3-carboxymethoxy-phenyl)-2-(4-sulfophenyl)-2H-tetrazolium] (MTS), 4-[3-(4-iodophenyl)-2-(4-nitrophenyl)-2H-5-tetrazolio]-1,3-benzene disulfonate (WST-1) and 3-(4,5-dimethylthiazol-2-yl)-2,5-diphenyltetrazolium bromide (MTT) can be mentioned. These dyes are reduced by viable cells, and this reduction is measured to calculate parameters such as mean cytotoxic concentration (CC50) and mean inhibitory concentration (IC50) [99,100].

Regarding the evaluation of antiviral activities, cells are infected with the virus, followed by treatment with nanoformulations. The efficacy of the nanosystems is measured through viral titer analysis, and calculations such as mean effective concentration (EC50) can be performed [101,102].

Among the articles found that developed nanoparticles, we can mention the work of Donalisio et al. [14], who produced nanodroplets coated with chitosan and cyclodextrin (SBEβCD) containing VACV. Cytotoxicity analysis was performed using the MTS assay on Vero cells (African green monkey kidney cells). The results showed that all tested compounds exhibited a mean cytotoxic concentration above 90 µM. Thus, all experiments were conducted using safe concentrations to avoid any possible inhibitory activity induced by treatment with the nanoformulations. To evaluate the antiviral activity of the nanoformulations, Vero cells infected with HSV-2 were used. Free valacyclovir showed an EC50 of 0.98 µM. The assays performed with the drug encapsulated in the nanodroplets showed a significant increase in antiviral capacity. Nanodroplets without SBEβCD achieved an EC50 of 0.43 µM, while those containing SBEβCD had an EC50 of 0.26 µM, confirming the greater efficacy of the system modified with SBEβCD [14].

In the study by Sangboonruang et al. (2022) [40], the cytotoxicity of PLA nanoparticles containing propolis extract was evaluated in Vero cells using the trypan blue exclusion assay. No cytotoxicity was observed at concentrations between 0 and 1.25 mg/mL. However, cell viability decreased considerably at a concentration of 2.5 mg/mL. Therefore, the evaluation of antiviral activity was performed using concentrations below 2.5 mg/mL. Infected cells were treated with the nanoparticles, and it was observed that the propolis-containing nanoparticles exhibited dose-dependent inhibition, with an IC50 of 0.80 ± 0.16 mg/mL. The empty nanoparticles showed no effect [40].

Mariotti et al. (2024) [38] tested their PLGA nanoparticles modified with a monoclonal antibody in Vero cells to assess possible cytotoxicity. No cytotoxic effects were observed in the cell cultures in the presence of the nanoparticles, confirming the safety of the developed systems [38].

In the study by Lima et al. (2018) [41], the viability of Vero cells was evaluated after exposure to PLA nanoparticles with and without chloroquine using the MTT assay. The nanoparticles without the drug showed no cytotoxicity, whereas the free drug exhibited dose-dependent toxicity. No significant toxicity was observed in cells treated with concentrations below 62.5 µg/mL. The drug-loaded nanoparticles were tested at dilutions ranging from 70 µg/mL to 2.5 µg/mL. Similar to the free drug, the chloroquine-loaded nanoparticles exhibited dose-dependent toxicity. The CC50 for the free drug was 222.6 µg/mL, and for the encapsulated drug it was 67.9 µg/mL. Cell viability remained above 80% for tested concentrations below 30 µg/mL. Therefore, concentrations ranging from 30 µg/mL to 2.5 µg/mL were used to evaluate antiviral activity. Vero cells were infected with Herpes simplex virus type 1 and treated with the nanoparticles to assess antiviral activity. The empty nanoparticles showed no antiviral effect. The IC50 for chloroquine was 6.7 µg/mL, while for the drug-loaded nanoparticles it was 4.3 µg mL^−1^, revealing the higher antiviral efficacy of the nanoparticle formulation [41].

In the study by Steinbach et al. (2012) [36], cell viability was determined in HeLa cells (human cervical cancer cells) treated with PLGA nanoparticles containing siRNA using the Cell Titer Blue assay. According to the results, the nanoparticles showed no cytotoxicity. To confirm the activity of siRNA in reducing messenger RNA (mRNA) expression, real-time PCR was performed to quantify mRNA levels. It was observed that the nanoparticles contributed to the decreased expression of nectin in HeLa cells [36].

PEGylated polystyrene nanoparticles were used in cervicovaginal mucus pretreated with Pluronic F-127 in Ensign et al. (2014) [34] studies. The transport behavior of HSV was observed to determine whether the barrier properties were compromised. The behavior of these nanoparticles was evaluated to assess whether the use of Pluronic could effectively enhance the penetration of nanosystems. Non-PEGylated nanoparticles were highly adhesive to the cervicovaginal mucus, whereas the PEG-modified particles diffused rapidly. When the mucus was treated with 0.01% Pluronic, the non-PEGylated nanoparticles remained immobilized. However, when treated with a 1% Pluronic solution, these nanoparticles diffused at rates similar to those of the PEG-containing nanoparticles. The displacement of non-PEGylated nanoparticles in cervicovaginal mucus pretreated with 1% Pluronic F-127 was significantly greater than in untreated mucus or mucus treated with 0.01% Pluronic. Nevertheless, this difference was not significant compared to the PEG-containing nanoparticles. In additional experiments, the diffusion of the herpes simplex virus was compared in mucus at pH 7 and in mucus pretreated with Pluronic F-127. In the neutralized mucus samples (pH 7), two viral populations were observed: one immobilized and another that diffused rapidly. In the mucus samples pretreated with Pluronic, the virus remained adhesively immobilized. These results demonstrated that treatment with Pluronic F-127 enhanced the penetration of nanoparticles into cervicovaginal mucus, potentially improving the delivery of encapsulated drugs [34].

In the study by Fayaz et al. (2012) [45], assays were conducted to evaluate the antiviral activity and cytotoxicity of condoms coated with silver nanoparticles. The WST-1 assay was used to assess the growth of HeLa cells, 293T cells (human embryonic kidney cells), and C8166 T cells (lymphoid cell line) after exposure to the nanoparticles. To evaluate antiviral activity against HSV, the nanoparticles were tested in Vero cells. The cytotoxicity assays showed that treating HeLa cells with polyurethane coated with silver nanoparticles did not affect cell growth after 96 h of culture. Similarly, the growth of 293T and C8166 T cells was not affected after 4 days of culture. Thus, it was concluded that the nanoparticles did not affect the viability of the tested cells. In the experiments performed to assess antiviral activity, it was observed that after exposure to polyurethane without silver nanoparticles, viral infectivity remained effective for the herpes simplex virus. In contrast, when the virus was exposed to polyurethane coated with silver nanoparticles, infectivity was completely lost, demonstrating the antiviral efficacy of the developed system [45].

In the study by Krzyzowska et al. (2022) [51], the cell viability of Vero cells, HaCaT cells (human keratinocyte cells), and VK-2-E6/E7 cells (human vaginal epithelial cells) treated with silver and gold nanoparticles was analyzed using the MTT assay. It was observed that the nanoparticles were slightly more toxic to VK-2-E6/E7 cells. Based on the obtained results, the appropriate nanoparticle concentrations were determined for use in antiviral activity assays, ensuring that cytotoxic concentrations were not reached. The antiviral activity was evaluated in Vero cells infected with HSV-2. Incubating the virus with lactoferrin-modified nanoparticles prior to infection demonstrated size-dependent viral inactivation. Silver nanoparticles were more effective than the gold-modified ones, with the 30 nm silver nanoparticles showing the highest antiviral activity. Unmodified nanoparticles did not show significant inhibition of viral infection in the cells. The test with a lactoferrin solution showed significant viral inhibition. This compound is known to be a glycoprotein with a potent inhibitory effect on the Herpes simplex virus by preventing viral binding and entry into host cells [51].

In the study by Krzyzowska et al. (2023) [47], the MTT assay was performed to evaluate cell viability in Vero, HaCaT and VK-2-E6/E7 cells treated with silver nanoparticles modified with epigallocatechin gallate (EGCG) and tannic acid (TA). The EGCG-AgNPs showed lower cytotoxicity compared to the unmodified nanoparticles. Antiviral activity was assessed in Vero cells infected with HSV-1 and HSV-2. Incubation of HSV-1 and HSV-2 with EGCG-AgNPs and TA-AgNPs for one hour before infection resulted in significant inhibition of viral infectivity. The unmodified nanoparticles did not show significant inhibition of infection [47].

In the study by Halder et al. (2018) [49], cytotoxicity assays were conducted in Vero cells and evaluated using the MTT assay. The results showed that gallic acid-stabilized gold nanoparticles exhibited a higher CC50 (972.4 ± 11.6 μM) compared to free gallic acid (786.5 ± 8.4 μM). Antiviral efficacy was determined in infected Vero cells, where a dose-dependent reduction in plaque formation was observed. The nanoparticles showed an EC50 of 32.3 ± 1.8 μM and 38.6 ± 2.9 μM for Herpes simplex virus types 1 and 2, respectively. Free gallic acid presented an EC50 of 80.2 ± 3.1 μM and 78.5 ± 4.8 μM for types 1 and 2, respectively [49].

The data from the in vitro assays, as well as other examples of developed nanoparticles, can be found in Table 1.

In a study by Klimova et al. (2020) [54], water-soluble fullerene C60 derivatives were developed to evaluate activity against HSV-1. An inhibition of approximately 90% was observed when compared to the acyclovir control (which inhibited only approximately 40%) [54]. The same was observed in a study by Fedorova et al. (2012) [55], in which fullerene C60 derivatives showed greater antiviral activity and lower cytotoxicity [55].

Regarding the articles related to liposomes/niosomes, the work by Jbara-Agbaria et al. (2022) [16] can be highlighted. They developed liposomes for encapsulating siRNA (siHSV) to inhibit the gene expression of the infected cell protein 0 (ICP0). The cytotoxicity of the liposomes was evaluated in SMC cells (smooth muscle cells) and HaCaT cells. The results showed that the liposomes were non-cytotoxic to SMC cells but exhibited moderate toxicity in HaCaT cells. Antiviral activity was assessed in HaCaT cells, and ICP0 expression levels were also analyzed. Treatments with liposomes containing siHSV and with free siHSV both demonstrated high antiviral activity. Additionally, a reduction in ICP0 levels was observed [16].

Krylova et al. (2022) [18] developed liposomes encapsulating antiviral carrageenan and echinochrome, evaluating them in Vero cells. Cytotoxicity MTT was low for carrageenans (CC50 > 1000 µg/mL) and liposomes (CC50 > 2000 µg/mL), but higher for echinochrome (CC50 142 µg/mL). In antiviral assays, free carrageenan/echinochrome complexes were more potent in virus pre-treatment (virucidal), with IC50s 20× (κ complex: 3.7 vs. 80 µg/mL) and 7× (Σ-complex: 2.8 vs. 20 µg/mL) lower than the compounds alone. In simultaneous treatment, complexes were also 3.5–4.7× more inhibitory. In cell pre-treatment (prophylaxis), κ-carrageenan was effective. Post-infection treatment showed moderate inhibition. The Σ-carrageenan/echinochrome complex was the most active, acting at early stages (viral binding). Comparing the free Σ-complex to its liposomal form: the free complex was more virucidal (IC50 2.8 vs. 9.5 µg/mL), but the liposome had higher prophylactic activity (cell pre-treatment) and effectively reduced plaques in post-infection treatment. In simultaneous treatment, both showed high activity with no significant difference [18].

In the study by Parsa et al. (2014) [58], cell viability was assessed in HeLa cells in the presence of niosomes containing acyclovir using the MTT assay. The results showed no apparent cytotoxicity for the tested formulations. Antiviral activity was evaluated in infected HeLa cells. The IC50 for the niosomes was 1 μM, while for the free drug it was 3 μM. This demonstrates the higher antiviral efficacy of the niosomes, which can be attributed to greater interaction of the system with the cells [58].

In the study by Szymanska et al. (2022) [62], the cytotoxicity of the formulations was evaluated in VK2/E6E7 cells (human vaginal epithelial cells) using the MTT assay and the JC-1 MitoScreen kit (cationic dye 5,5′,6,6′-tetrachloro-1,1′,3,3′-tetraethyl-benzimidazolylcarbocyanine iodide). No cytotoxic effects were observed in either assay. Antiviral activity was assessed in infected VK2/E6E7 cells. Greater viral inhibition was observed in cells treated with the nanofibrous formulations. However, viral inactivation did not increase with higher concentrations of nanofibers, likely due to reduced drug uptake and saturation of enzymes critical for prodrug hydrolysis and activation [62].

### 3.7. In Vivo Studies

In vivo experiments provide fundamental information on bioavailability, metabolism, systemic toxicity and potential immune effects. These studies represent an essential step in the preclinical development of new drug delivery systems, as they allow for an integrated assessment of therapeutic efficacy, safety and the organism’s biological response to the developed formulation.

These investigations are primarily conducted in mice and rats, as they are biological models with ease of handling, accessible cost, and physiological responses similar to humans in viral infections. Furthermore, they make it possible to visually and quantitatively monitor the progression of cutaneous or genital lesions, which are common in infections caused by HSV-1 and HSV-2 [101,102].

A total of 35 articles were found and added to Table 1, with only 14 being in vivo studies, as shown in Table 2. These studies represent the most advanced stage of research seeking to apply nanomaterials in the treatment of herpetic infections. The central objective of most of them was to enhance the efficacy and safety of antivirals already in use, such as acyclovir, or to explore new bioactive molecules with antiviral potential, using nanocarriers and nanoformulations capable of increasing the stability, penetration and duration of action of the substances.

Among the most frequently used systems, polymeric nanoparticles (such as PLGA and chitosan), solid lipid nanoparticles (SLNs) and metallic nanoparticles (such as gold, selenium and zinc oxide) stood out. Each type of nanoparticle was chosen according to specific properties, such as biocompatibility, mucoadhesion, controlled release and the ability to stimulate antiviral immune responses. The studies that presented in vivo assays were: 6 for polymeric/lipid nanoparticles, 4 for inorganic/metallic/composite nanoparticles, 1 for fullerene/carbon nanodots, 1 for liposomes, and 2 for nanoemulsions.

Ensign et al. (2014) [34] evaluated the effect of Pluronic F127 on cervicovaginal mucus (CVM) in murine models. Daily vaginal applications of a 1% solution were monitored for one week, assessing inflammatory cytokines (IL-1α and IL-1β) and epithelial integrity. The Pluronic did not increase inflammation or alter the mucosal barrier, unlike Nonoxynol-9. Furthermore, it facilitated nanoparticle penetration and distribution without increasing susceptibility to infection, demonstrating it to be a safe agent for intravaginal applications [34].

Steinbach et al. (2012) [36] developed PLGA nanoparticles for the delivery of siRNA targeting essential HSV-2 genes in a murine model of genital infection. The encapsulated siRNA was administered intravaginally in prophylactic and therapeutic regimens. The formulations were evaluated for viral load by qPCR, titration in vaginal swabs, clinical score and target gene expression by RT-qPCR. The siRNA-containing nanoparticles promoted a significant reduction in viral replication, a delay in clinical progression, and lower symptom severity compared to free siRNA. Encapsulation increased the stability and tissue retention time of the siRNA, resulting in greater delivery efficiency. No biochemical alterations or signs of systemic toxicity were observed, confirming the formulation’s safety profile [36].

Shen et al. (2019) [43] developed *Rheum tanguticum* nanoparticles and evaluated the in vivo effects in BALB/c mice cutaneously infected with HSV-1. Topical applications of the NPs reduced the expression of immediate-early viral genes (ICP4, ICP8), decreased the viral load and increased the survival rate. Histology showed less inflammation and tissue degeneration. Furthermore, systemic toxicity was minimal. The work concluded that nanoencapsulation of phytotherapeutic compounds increases stability and antiviral potency [43].

Krzyzowska et al. (2022) [51] evaluated silver and gold nanoparticles functionalized with lactoferrin in BALB/c mice infected with HSV-2, both cutaneously and intravaginally. Different sizes and surface modifications were compared. The 30 nm silver particles showed the greatest antiviral effect, and conjugation with lactoferrin potentiated viral inactivation. There was also an induction of a local immune response and lower recurrence. The work highlighted the importance of size and functionalization in the efficacy of nanoparticles [51].

Antoine et al. (2016) [53] analyzed the effect of zinc oxide (ZnO) tetrapods as micro-nanocarriers in female mice with vaginal HSV-2 infection. Tests included isolated ZnO, standard antiviral, ZnO + antiviral combination and control. Analyses comprised viral titration, vaginal histology and dosing of cytokines and immune cells. The ZnO strongly reduced viral replication and inflammation, and the combination with the antiviral resulted in a synergistic effect, with greater NK cell recruitment and increased IFN-γ. The authors concluded that ZnO possesses direct viral action and immunomodulatory potential [53].

Klimova et al. (2020) [54] developed water-soluble fullerene C60 and evaluated its efficacy against HSV-1 in DBA/2J mice. Treatments were performed topically, and it was observed that animals treated with the fullerene nanodot-based formulation showed the fewest lesions and the fastest recovery compared to the controls [54].

In the work of Jbara-Agbaria et al. (2022) [16], liposomes containing siRNA (LipDOPE-siHSV) were tested in BALB/c mice. Based on the assays, no hepatotoxic reactions were detected in the animals treated with the liposomes. Furthermore, biodistribution assays revealed that the nanosystem preferentially accumulated in organs such as the liver and spleen [16].

Al-Subaie et al. (2015) [60] investigated an optimized acyclovir nanoemulsion, using both ex vivo rat skin and Wistar rats for pharmacokinetic assays. Initially, the skin was subjected to permeation tests in Franz diffusion cells, and subsequently, the formulation was applied topically to the animals in single and repeated doses for seven days. Three groups were compared: nanoemulsion, acyclovir gel and commercial cream. Analyses involved quantification of permeation over time, spectrometry of plasma acyclovir levels, clinical observation of cutaneous lesions and hepato-renal biochemical exams. The nanoemulsion showed an approximately 2-fold increase in ex vivo permeation compared to the gel and 1.5-fold compared to the cream. In vivo, the relative bioavailability reached 535.2% and 244.6% compared to the gel and cream, respectively. Histology indicated less local irritation and serum parameters remained normal, confirming good tolerability. Thus, the study demonstrated that nanoemulsion improves cutaneous absorption and systemic exposure without causing apparent toxicity [60].

Overall, the in vivo protocols consist of infecting the animals (via cutaneous or genital routes) and then applying the nanoformulation topically, orally or intraperitoneally. Efficacy was evaluated through parameters such as lesion reduction, decreased viral load, histological analysis and dosing of inflammatory cytokines. Nearly all studies reported greater antiviral efficacy and lower toxicity of the nanoformulations compared to conventional treatments.

## 4. Future Directions and Limitations

Despite the expressive advancements observed in preclinical studies, the clinical application of nanostructured formulations against HSV still faces several limitations. One of the main issues is the lack of methodological standardization among different research groups. Although many studies use similar animal models, there is a wide variation in experimental conditions, such as the viral strains employed, infective dose, type of formulation, route and frequency of administration, and treatment duration, which makes it difficult to establish direct comparisons or generalize the results obtained [14,57].

Another critical point is the insufficiency of detailed information regarding the physicochemical parameters of the nanoparticles, such as mean diameter, PDI, zeta potential and encapsulation efficiency. These factors are determinants for the stability, bioavailability and interaction of nanoparticles with biological tissues. In some articles, it was observed that the absence of data on the number of animals, inclusion and exclusion criteria, as well as the lack of randomization and blinding, could introduce experimental biases and compromise the reproducibility of the results. Furthermore, robust studies of pharmacokinetics, biodistribution and metabolism of nanoparticles in target organs, especially the liver, kidneys, spleen and central nervous system, are scarce. Understanding these aspects is essential for predicting possible cumulative toxic effects, residence time in the organism and eventual long-term bioaccumulation [40,51]. The lack of robust toxicological data also limits the extrapolation of results to humans, with the urge to perform acute, subchronic and chronic toxicity studies, both local and systemic. From a technological and production point of view, scaling up formulations developed at the laboratory level to larger industrial processes still represents a substantial challenge. Minor variations in synthesis conditions, such as temperature, pH, agitation rate or drying method, can significantly alter the critical properties of the nanoformulation, directly influencing its efficacy and safety [50]. This process sensitivity hinders large-scale reproducibility and demands rigorously controlled production protocols. It is also important to highlight that many studies focus on acute infection models, whereas HSV is characterized by its capacity for latency and recurrent reactivation. Few works have evaluated the ability of nanoformulations to prevent viral reactivation or act on latent reservoirs, which would be essential for developing long-term therapies or curative strategies.

Some studies have been found associating the use of photodynamic therapy with the treatment of lesions related to papillomavirus infections; however, little has been discussed regarding its use in the treatment of infections caused by HSV [94,103,104]. Photodynamic therapy may offer a less invasive and more effective treatment with a lower risk of toxicity. Some studies have already demonstrated the effectiveness of photodynamic therapy in reducing lesions in cases of herpes labialis. Treatment of the early stages using a photosensitizer has shown promising results; nevertheless, more rigorous clinical studies are still required to confirm the therapy’s efficacy [103,105,106,107]. Treatment with siRNA is an interesting option for treating various diseases such as cancer, autoimmune diseases, and viral infections. The therapy is in the development phase, and pharmaceutical nanotechnology may help promote the use of siRNA [108].

Even in the face of these limitations, the evidence we have obtained so far demonstrates a promising therapeutic potential for nanotechnology in treating herpetic infections. This clarifies the objective of consolidating reproducible methodologies and strengthening regulatory guidelines, which is expected in the near future, so that these formulations can be incorporated into clinical practice, offering more effective, safer and well-targeted therapies.

## 5. Conclusions

Herpes simplexes (HSV-1/HSV-2) are recurrent infections that are difficult to control, especially due to the virus’s ability to remain latent in the organism and reactivate upon specific stimuli or low immunity at different times. Furthermore, current treatments aid in management but do not completely prevent the appearance of new lesions. Another issue arises in cases of drug resistance, which consequently complicates the achievement of a momentary “cure.” Nanotechnology offers various models of nanosystems and nanocarriers to combat the herpes virus. In this context, nanotechnology emerges as a promising alternative for treating this viral infection without the need for frequent reapplications, thus reducing side effects.

Acyclovir and valacyclovir are generally well tolerated; however, individual variability and underlying clinical conditions, such as hepatic or renal impairment, circulatory disorders, and infectious comorbidities (HIV infection), may increase the risk of drug-related adverse effects. In this context, topical administration represents a valuable strategy to minimize systemic exposure and potentially reduce the incidence of adverse reactions while maintaining local antiviral activity. Moreover, a combined therapeutic approach integrating oral and topical delivery may be advantageous for managing severe or recurrent infections, enabling control of viral replication at the lesion site.

The analyzed studies show that different types of nanoparticles can increase the stability of antivirals, prolong their action, facilitate tissue penetration and even stimulate the immune system. Overall, both the in vitro and in vivo results were superior to those obtained with traditional drugs, presenting lower toxicity. However, before these formulations can be used clinically, more standardized studies, long-term safety analyses and the development of large-scale production methods are still necessary. In addition, current evidence suggests that nanotechnology holds significant promise for the future treatment of herpes simplex virus (HSV) infections. Nano-based delivery systems may enhance the therapeutic efficacy of antiviral agents by improving drug stability, bioavailability, and targeted delivery to infected tissues. Consequently, these approaches have the potential to accelerate symptom resolution, reduce viral load, and minimize patient discomfort, ultimately contributing to more effective and patient-friendly therapeutic strategies.

## Figures and Tables

**Table 1:** Nanosystem, mechanism, composition, characterizations and in vitro studies.

Ref.	Nanosystem/Mechanism	Composition	Drug/Active	Size (nm) PDI	Key In Vitro Results
**POLYMERIC/LIPID NANOPARTICLES**					
**[14]**	Chitosan NPs/Nanocarrier for Drug delivery	Chitosan nanoparticles are physically cross-linked with SBE-β-cyclodextrin, loading valacyclovir via cyclodextrin inclusion and polymer encapsulation.	Valacyclovir (Drug delivery)	252–400 0.2	Chitosan nanodroplets functionalized with sulfobutyl-ether-β-cyclodextrin (SBEβCD) and loaded with valacyclovir exhibited superior antiviral activity against HSV compared with the free drug. The formulation showed enhanced mucoadhesion, sustained release, and improved physical stability, suggesting better therapeutic efficiency at mucosal sites.
**[34]**	Polystyrene-PEG NPs and Pluronic F127 (Penetration enhancer)	Pluronic F127 (Penetration enhancer) to Polystyrene NPs functionalized with PEG.	Test particles (no drug) (Pluronic F127 acts as penetration enhancer of PS-PEG)	200 -	Pretreatment of cervicovaginal mucus (CVM) with the surfactant Pluronic F127 increased the diffusion of PS-PEG through the mucus layer without impairing its natural protective barrier against HSV, indicating enhanced nanoparticle mobility and improved potential for mucosal drug delivery.
**[35]**	Polyanionic carbosilane NPs (cationic dendrimer)/Virucide (physical interaction): viral inactivation through surface binding)	Polyanionic carbosilane (dendrimer: G2-S16)	G2-S16 carbosilane dendrimer (antiviral nanomaterial)	21.32 -	The G2-S16 dendrimer effectively inhibited HSV-2 infection in vitro, maintaining antiviral activity even in the presence of semen.
**[36]**	PLGA NPs/Nanocarrier for Drug delivery (Gene Therapy)	Polymeric (cationic) nanoparticles of PLGA for siRNA delivery targeting HSV-2.	siRNA (Biomolecules)	160–189 0.19	In vitro studies demonstrated that cationic polymeric nanoparticles effectively delivered anti-HSV-2 siRNA into infected cells, leading to strong viral gene silencing and a significant decrease in viral replication, while maintaining cell viability and ensuring efficient siRNA protection and intracellular delivery.
**[37]**	PLGA NPs/Nanocarrier for Drug delivery	PLGA nanoparticles loaded with *Cymbopogon citratus* volatile oil; incorporated into carbomer hydrogel.	Essential oil (Drug delivery for Natural product)	217 0.2	PLGA nanoparticles (Size: 217 nm) with negative surface charge were successfully incorporated into a Carbomer^®^ gel. The resulting nanogel showed a biphasic release profile. An initial burst release was followed by sustained delivery. Volatile components were protected from evaporation. Local drug availability was enhanced at the application site. The formulation inhibited HSV at low concentrations, indicating improved antiviral potency in comparison to essential oil-free.
**[38]**	PLGA-PEG-Bis-Sulfone NPs/Virucide (viral inactivation by surface link by monoclonal antibody) and diagnosis	PLGA-PEG-Bis-Sulfone nanoparticles covalently conjugated with anti-gG2 (HSV-2) Fab fragments for targeted antiviral activity.	Antibody targeting HSV-2 glycoprotein G2 (Immunonanoparticle)	151–10.4 0.3	Antibody-conjugated nanoparticles increased in size (approximately 151 ± 10.4 nm) and remained stable. Specific binding to the HSV-2 gG2 glycoprotein was confirmed by SPR with low micromolar affinity (approximately 1.03 µM). In vitro assays showed no significant cytotoxicity in Vero cells. Targeted recognition of HSV-2 was successfully achieved. Immunonanoparticles (conjugated with monoclonal antibodies) are suitable for early diagnosis and treatment of the viral infection.
**[39]**	Benzhydryl amide core NPs (dendrimer)/Virucide (physical interaction): Inactivation by binding of the monoclonal antibody to the viral surface	NPs based in Polymeric dendrimers with benzhydryl amide core, lysine branches, and anionic surface groups (DNAA or 3,5-disulfobenzoic acid).	SPL7013 and SPL7115 Dendrimers (antiviral nanomaterials)	200 -	G3 DNAA dendrimer (EC_50_ = 0.12 ± 0.02 mM); G3 3,5-Ph-(SO_3_Na)_2_ dendrimer (EC_50_ = 0.26 ± 0.08 mM); G3 4-Ph-SO_3_Na dendrimer (EC_50_ = 19.36 ± 4.8 mM). Dendrimers inhibited HSV-2 infection in HEL cells without cytotoxicity. DNAA- and 3,5-Ph-(SO_3_Na)_2_-capped dendrimers showed the highest antiviral potency, while 4-Ph-SO_3_Na dendrimers were ~160-fold less potent. Antiviral activity correlated with higher anionic charge density and hydrophobicity, indicating inhibition at the viral entry stage.
**[40]**	PLGA NPs/Nanocarrier for Drug delivery	Ethanolic extract of propolis encapsulated in PLGA NPs; evaluated against HSV-2.	Propolis actives (Drug delivery for natural product)	450 0.21	Evaluations of polymeric nanoparticles encapsulating ethanolic extract of propolis (EEP-NPs) revealed potent anti-HSV-2 activity, primarily through the inhibition of viral entry and replication. The formulation exhibited good biocompatibility and safety for mucosal use, indicating its potential as a natural, nanoparticle-based antiviral approach.
**[41]**	PLA NPs/Nanocarrier for Drug delivery	PLA polymeric nanoparticles designed to encapsulate hydrophilic chloroquine diphosphate.	Chloroquine diphosphate (Drug delivery)	<300 0.3	PLGA nanoparticles loaded with chloroquine diphosphate significantly enhanced antiviral activity against HSV-1. Superior effectiveness was linked to sustained release and increased cellular internalization. The formulation markedly reduced viral replication. Cell viability was better preserved compared to free chloroquine. These results highlight the potential of PLGA nanoparticles to boost antiviral efficacy.
**[42]**	PCL-MPEG NPs/Nanocarrier for Drug delivery	PCL-MPEG NPs for controlled release of acyclovir.	Acyclovir	<200 -	Amphiphilic polymeric micelles self-assembled for drug loading. In vitro studies demonstrated that amphiphilic polymeric micelles efficiently encapsulated acyclovir, enhancing its solubility and providing sustained release over time. The micellar formulation improved cellular uptake of the drug and prolonged its antiviral effect compared with free acyclovir, while showing minimal cytotoxicity, indicating effective delivery without compromising cell viability.
**[43]**	Nanoparticles of Plant extract/Nanocarrier for Drug delivery	Plant-based nanoparticles derived from *R. tanguticum* extract; antiviral assays in vitro and in vivo against HSV-1.	*R. tanguticum* extract with natural molecules (Natural product)	50–219 0.13	Nanoparticles derived from *Rheum tanguticum* extract effectively inhibited HSV-1 replication.
**[44]**	Lipid NPs/Nanocarrier for Drug delivery	SLPNs (Solid Lipid Nanoparticle) optimized using response surface methodology; lipid: Biogapress Vegetal 297 ATO and Tween 80.	Acyclovir	130 0.22	SLPs loaded with acyclovir, formulated with Biogapress Vegetal 297 ATO and stabilized with Tween 80, maintained high drug encapsulation and provided sustained release over time. The formulation enhanced acyclovir uptake by cells and prolonged antiviral activity compared with free drug. Cytotoxicity assays confirmed good cellular tolerance, demonstrating that the SLN system delivered the drug efficiently without affecting cell viability.
**INORGANIC/METALLIC/COMPOSITE NANOPARTICLES**					
**[28]**	PoSeNPs/ Virucide (physical interaction): glycoprotein agglutination and preventing binding between the virus and the host cell	Green synthesis of *Polycladia myrica* aqueous extract-Selenium NPs.	*Polycladia myrica* and Selenium	17–23 0.094	PoSeNPs were uniform, small (~17–23 nm), and biocompatible at tested concentrations. They showed in vitro antiviral activity against HSV-1, reducing infectivity and viral replication. Low cytotoxicity was detected in healthy cells. These results indicate a therapeutic window for combined antivirals. PoSeNPs have the ability to adhere to glycoproteins found on the surface of the virus glycoprotein agglutination and preventing binding between the virus and the host cell.
**[45]**	AgNPs Virucide (physical interaction): viral inactivation through surface binding)	Synthesis of AgNPs (chemical/green variant described). AgNPs were uniformly coated on latex condom surface.	Silver	30–60 -	Showed that latex condoms coated with silver nanoparticles (AgNPs) achieved effective inactivation of HIV and HSV by blocking viral attachment and entry. The coating maintained the mechanical integrity of the latex material, supporting its feasibility for antiviral barrier protection.
**[46]**	AgNPs Virucide (physical interaction): viral inactivation through surface binding)	Biosynthesized using fungi (mycosynthesis); tested against HSV-1 and HSV-2.	Silver	20–50 <0.2	Fungus-mediated silver nanoparticles (AgNPs) revealed broad-spectrum antiviral effects, notably against HSV-1 and HSV-2 and human parainfluenza virus type 3. The biogenic AgNPs were stable, environmentally friendly, and maintained high antiviral potency without evident cytotoxicity.
**[47]**	AgNPs Virucide: viral inactivation through surface binding)	Silver nanoparticles surface-modified with EGCG; antiviral against HSV-1 and HSV-2.	EGCG-Modified Silver	30–35 -	Findings showed that silver nanoparticles functionalized with epigallocatechin gallate (EGCG–AgNPs) effectively inhibited HSV-1 and HSV-2 infection by preventing viral binding and cellular entry. The system also provided synergistic antioxidant and antiviral benefits, supporting its relevance as a multifunctional therapeutic platform.
**[48]**	AgNPs Virucide (physical interaction: viral inactivation through surface binding)	Silver nanoparticles synthesized through chemical redox method	Silver	10 -	Demonstrated that chemically synthesized silver nanoparticles of varying sizes and surface properties inhibited HSV-1 infection by binding to the viral surface and preventing entry into host cells. The antiviral effect was size-dependent, with smaller nanoparticles showing stronger inhibition, while maintaining low cytotoxicity toward the host cells.
**[49]**	AuNPs Virucide (physical interaction: viral inactivation through surface binding)	Monodisperse gold nanoparticles (AuNPs) fully physiochemically characterized and tested against HSV.	Gold	18.27 0.14	Experiments showed that highly monodispersed gold nanoparticles, synthesized via controlled reduction, effectively inhibited HSV infection in cultured cells. AuNPs are able to bind to the viral surface, preventing infection of the host cell. The nanoparticles displayed uniform size and shape, promoting consistent antiviral activity while exhibiting minimal cytotoxicity, confirming their safety and efficiency at the cellular level.
**[50]**	AuNPs Virucide (physical interaction: viral inactivation through surface binding)	Functionalized AuNPs crossing the blood–brain barrier (BBB) to prevent HSV-1 infection and reduce amyloid-β secretion.	Gold	10–500 0.1	The results showed that functionalized gold nanoparticles equipped with BBB–penetrating ligands effectively prevented HSV-1 infection in neuronal cells. The treatment also reduced amyloid-β secretion, suggesting both antiviral activity and neuroprotective potential, while exhibiting minimal cytotoxicity.
**[51]**	AgNPs or AuNPs/Virucide (immunomodulatory action) (physical interaction: viral inactivation through surface binding)	Silver and gold NPs functionalized with Lactoferrin; tested as antiviral/immunomodulatory agents.	Lactoferrin-modified Silver-Gold	20–40 -	Demonstrated that silver and gold nanoparticles functionalized with lactoferrin (LF–AgNPs and LF–AuNPs) displayed potent antiviral activity against HSV-2. These nanoconjugates also modulated immune responses at the mucosal surface, indicating a combined antiviral and immunoprotective potential.
**[52]**	ZnO NPs/Virucide: generation of Reactive Oxygen Species (ROS), interaction and damage to the viral structure, dissolution and release of zinc ions (Zn^2+^) toxic to the virus	Green synthesis of ZnO NPs using *P. indica* extract; antiviral activity against HSV-1.	Zinc oxide	23–35 -	Analyses showed that ZnO nanoparticles synthesized using *Plumbago indica* L. extract strongly suppressed HSV-1 replication. Antiviral mechanism involved disruption of viral attachment and a decrease in infectivity, achieved without inducing cytotoxic effects in host cells.
**[53]**	Tetrapod-shaped ZnO NPs Immunomodulation: activation of the immune system against viral antigen Virucide: nanoparticles blocking viral attachment in the cell surface and entry.	Tetrapod-shaped ZnO nanoparticles for intravaginal immunoprotection against genital herpes.	Modified Zinc oxide	200 -	There are no in vitro studies, only in vivo studies (Table 2).
**FULLERENE/CARBON NANODOTS**					
**[54]**	Fullerene (dnC60)/Virucide (physical interaction): nanomaterial binding to viral surface preventing the virus from interacting with the cellular receptor, blocking its entry into the cell	Water-soluble Fullerene dnC60 and adducts	Fullerene C60 and adducts (antiviral nanomaterial)	<100 -	All derivatives suppressed HSV-1 infection using the pretreatment assay. The strongest effect was observed for dnC60, which achieved an inhibition of 87% while ACV inhibited only 37%. Only dnC60 and ACV were effective in reducing HSV-1 infection, with inhibition rates of 60% and 93%, respectively. In addition, dnC60 was the derivative that showed antiviral activity, inhibiting HSV-1 infection in Vero cells by 98.2% in the virucidal assay. The other compounds and the positive control (ACV) did not show virucidal activity or exhibited little activity against HSV-1.
**[55]**	Fullerene-based carbon NPs/Virucide (physical interaction): viral inactivation through Fulerene viral particle surface binding	Water-soluble carboxylic fullerene C60 derivatives (polycarboxylated C60 potassium salts)	Fullerene C60 (antiviral nanomaterial)	~100 0.2	Fullerene C60 derivatives (EC50 range: 0.52–23 µg/cm^3^ for HSV; 0.57–17 µg/cm^3^ for HCMV, depending on derivative). Several fullerene derivatives exhibited strong antiviral activity against HSV and HCMV with low cytotoxicity. Compound 2a showed the highest anti-HSV activity (EC50 = 0.52 µg/cm^3^; CTI = 1961), while compound 3 also showed high anti-HSV efficacy (CTI = 1796). Compound 2d demonstrated notable anti-HCMV activity (CTI = 127).
**[56]**	Carbon nanodots (C-dots)/ Virucide (physical interaction): viral inactivation through C-dots viral particle surface binding	Carbon nanodots synthesized by modified hydrothermal carbonization and surface-functionalized with 4-aminophenylboronic acid (4-AB/C-dots) or phenylboronic acid (B/C-dots)	Carbon nanodots (antiviral nanostructure)	96–332 <0.3	Carbon nanodots (EC50 = 80 ng/mL in Vero cells; 145 ng/mL in A549 cells). 4-AB/C-dots effectively inhibited HSV-1 infection at nanogram per milliliter concentrations, whereas B/C-dots showed no antiviral activity even at high concentrations. The nanoparticles interfered with early stages of viral entry, likely through interactions with the virus and host cells.
**LIPOSOMES/NIOSSOMES**					
**[16]**	Liposomes/Nanocarrier for Drug delivery (Gene therapy)	siRNA; modified thin film hydration by a modified ethanol injection technique	siRNA (Biomolecule)	100–200 <0.2	Efficient internalization by HaCaT cells without cytotoxicity; significant antiviral activity in plaque reduction assays and 3D epidermis model; reduced ICP0 expression.
**[18]**	Liposomes/Virucidal, Prophylactic properties and ability to inhibit virus_cell interaction	κ-CRG/Ech and Σ-CRG/Ech complexes and liposomal form of Σ-CRG/Ech	Carrageenan (κ- and Σ-) and Echinochrome A (Ech)	<500 -	Σ-CRG/Ech liposomal exhibited strongest antiviral activity against HSV-1 and HSV-2 in Vero cells; low cytotoxicity, high selectivity; mainly inhibited viral entry; partial virucidal effects; superior effectiveness and reduced toxicity compared to Σ-CRG/Ech complexes.
**[57]**	Liposomes/Nanocarrier for Drug delivery	Derivatives of 2-aminomethyl-3-hydroxy-1,4-naphthoquinone, with n-butyl, benzyl and nitrobenzene substituents; solvent evaporation and lipid layer hydration method	Naphthoquinone derivative	100 -	Inhibited early and late stages of HSV-1 replication; benzyl and nitrobenzene derivatives had ~9x higher selectivity index than acyclovir; liposomal encapsulation enhanced antiviral potency in comparison with free drug.
**[58]**	Niosomes/Nanocarrier for Drug delivery	Nonionic surfactant (Span), cholesterol and TPGS; film hydration method.	Acyclovir	123 -	Low cytotoxicity in HeLa cells; ACV-loaded niosomes showed greater antiviral activity than free acyclovir.
**NANOEMULSIONS**					
**[59]**	Nanoemulsions/Nanocarrier for Drug delivery (permeation enhancer)	Nanoemulsion containing Tween 20, Triacetin or Tramsectol^®^P were developed using pseudo-ternary phase diagrams	Acyclovir	15 -	Nanoemulsions were stable and exhibited sustained drug release. Nanoformulations F1 and F2 increased skin permeation of acyclovir by 2.85 and 2.90, respectively, compared to the control.
**[60]**	Nanoemulsions/Nanocarrier for Drug delivery (permeation enhancer)	Clove oil, castor oil, Tween 80, Span 80, propylene glycol e Myo-6V; spontaneous emulsification method	Acyclovir	43 -	After 2.5 h, cumulative ACV permeation reached 2.21 µg/cm^2^ for ACV-NE hydrogels, compared with 1.40 µg/cm^2^ for the marketed cream. The ACV-NE hydrogel showed a twofold increase in permeation versus the control gel (ACV hydrogel) (Permeation of 1.05 µg/cm^2^). Permeation was also 1.5-fold higher than the marketed cream. Less than 10% of ACV was recovered overall. This low recovery was attributed to drug retention within skin layers.
**[61]**	Nanoemulsions/Nanocarrier for Drug delivery (permeation enhancer)	Nanoemulsion obtained by emulsifying spontaneously prepared with Garlic Oil, Tween 20, Span 20, Propylene Glycol	Acyclovir	170	Transdermal nanoformulation increased the cutaneous permeation of acyclovir by approximately 2.3 times compared to the control.
**NANOFIBERS/NANOWIRES**					
**[62]**	Nanofibers/Nanomaterial for Drug delivery	Chitosan (CS)/Poly(ethylene oxide) (PEO) nanofibrous material produced by solution blow spinning (SBS)	Tenofovir disoproxyl fumarate	403 -	TDF-CS/PEO-based nanofibers are safe according to cytotoxicity studies and have shown in vitro antiviral effect against the HSV-2 virus. Furthermore, the SBS technique did not alter the virucidal action of the drug. Changes in vaginal pH (3.8–5.0) significantly affected the mucoadhesive and swelling properties of the nanofiber, influencing the release, permeation, and retention of TDF in vaginal tissue. An eventual increase in vaginal pH to 5.0 promotes greater permeation of TDF, improving bioavailability.
**[63]**	Nanowires (tin oxide)	Tin oxide (SnO_2_) nanowires were produced by flame transport synthesis approach	Not drug-loaded—antiviral effect by physical/chemical blockade of viral attachment.		SnO_2_ nanowires inhibited viral infection, reducing viral entry by approximately five-fold compared with the control. In addition, the nanowires decreased plaque size by 77% and reduced the number of infected clusters by 75%, inhibiting virus-induced cell–cell fusion by more than 99%. Importantly, no cytotoxic effects were observed at concentrations up to 3000 µg/mL, showing adequate safety profile.

Notes: NPs (Nanoparticles), PS-PEG (Polystyrene-polyethylene glycol), PCL (Poly-caprolactone), PLA (Poly-lactic acid), PLGA (Poly-lactic-co-glycolic acid), siRNA (small interfering RNA), EGCG (Epigallocatechin Gallate), SLPNs (Solid Lipid Nanoparticle), SBEβCD (Sulfobutyl-ether-β-cyclodextrin), F127 (Pluronic F123), DNAA (naphthalene disulfonic acid), EEP (Ethanolic extract of propolis), MPEG (Methoxy Polyethylene Glycol), PoSeNPs (Polycladia myrica aqueous extract-Selenium), BBB (blood–brain barrier), CRG (Carrageenan), Ech (Echinochrome A), TPGS (d-α-Tocopheryl Polyethylene Glycol 1000 Succinate), CS (Chitosan), PEO (Poly(ethylene oxide)), SBS (Solution blow spinning).

**Table 2:** Description and results of in vivo tests.

Ref.	Nanosystem	Animal Model	Key Tests Performed	Treatment Details	Groups/Controls	Key Results
			**POLYMERIC/LIPID NANOPARTICLES**			
**[34]**	Polystyrene-PEG NPs and Pre-treatment of the CVM with Pluronic F127 (Penetration enhancer)	Female CF-1 mice	Pluronic F127-Pretreated CVM aiming Penetration enhance/PS-PEG NPs In Vivo Cytokine Release (IL-1α, IL-1β)	Daily vaginal 1% Pluronic F127 for one week. Pharmacological study	Mice with mucus infected with viral particles were tested without pretreatment and with pretreatment using surfactant (Pluronic F127). In addition, PS-PEG NPs were administered.	HSV-1 remains attached to the mucous membrane (MCV) pre-treated with F127. Surfactant did not reduce viral particle-mucus interactions. There was no increase in the release of inflammatory cytokines in the vaginal tract of mice after daily application of 1% F127 for 1 week. The antimicrobial barrier of the vaginal mucosa was not altered by administration of 1% Pluronic F127, but it improved the distribution and penetration of NPs. Drug release in the vaginal epithelium may be improved by pre-treatment of MCV with the surfactant, which enhances the penetration and distribution of NPSs.
**[35]**	Polyanionic carbosilane NPs (cationic dendrimer)/Virucide (physical interaction): viral inactivation through surface binding)	Female BALB/c mice	Gel containing dendrimer is able to prevent viral infection by inactivating the viral particle.	Vehicle is a hydroxyethyl-cellulose (HEC) gel and the active pharmaceutical is the (G2-S16) cationic dendrimer (nanoparticle). Biological study	Four groups of 3 mice per group: group control with no treatment (group NT), group treated with G2-S16 dendrimer, group infected by HSV-2, and group treated with G2-S16 dendrimer and post-infected by HSV-2.	Dendrimer G2-S16 has a protective effect against HSV-2 infection by preventing viral entry and maintaining the composition of the vaginal microbiome.
**[36]**	PLGA NPs/Nanocarrier for Drug delivery of siRNA (gene therapy)	Female C57BL/6 mice	Nanoparticles (NPs) are capable of releasing siRNA with the aim of preventing viral infections.	siRNA loaded in NPs was administered to the vaginal mucosa with an administration schedule. Pharmacological study	Control group without viral infection. Group infected with the virus without treatment. Group infected and treated with free siRNA and encapsulated in NPs.	In vivo antiviral efficacy was confirmed with intravaginal administration of siRNA-loaded nanoparticles using a murine model of HSV-2 infection. The topical nanoformulation was able to prevent viral replication and infection.
**[39]**	Benzhydryl amide core NPs (dendrimer)/Virucide (physical interaction): Inactivation by binding of the monoclonal antibody to the viral surface	Female CF-1 mice	In vivo toxicity and HSV-2 inhibitory activity were evaluated in the mouse HSV-2 susceptibility model. Dendrimer based on Benzhydryl amide is able to prevent viral infection by inactivating the viral particle.	Vehicle is gel (Vivagel^®^, Aspen Pharmacare, Melbourne, Australia) and the active pharmaceutical is the dendrimer. Intravaginal administration of the nanoformulation. Biological study	Two groups: treated with dendrimer (Vivagel^®^) and post-infected by HSV-2.	The nanoformulation provides prolonged in vivo protection against HSV-2 in an animal model of vaginal transmission. Furthermore, the nanoformulation is biocompatible with the vaginal mucosa.
**[43]**	Nanoparticles of Plant extract/Nanocarrier for Drug delivery	Male Kunming mice	*Rheum tanguticum* nanoparticles were administered by gavage into the rats infected with the virus.	The inoculated mice received the following treatment: *R. tanguticum* nanoparticles were administered at 625 312.5 mg/kg/day, respectively; the positive controls received 100 mg/kg/day acyclovir. Saline (0.9%) was used in normal and viral controls. The drugs were administered via oral gavage once daily or acyclovir twice daily at 12 h intervals for five consecutive days Pharmacological study	Five groups and inoculated intracerebrally containing either no virus (vehicle only) or LD_50_ of HSV-1. Saline (0.9%) was used in normal and viral controls.	NPs were able to protect mice against the lethality of viral brain infection. There was a reduction in viral load and protection against brain tissue damage. In vivo findings confirmed a reduction in viral load and lower levels of inflammatory cytokines in infected models, supporting the dual antiviral and anti-inflammatory properties of the formulation.
**[44]**	Lipid NPs/Nanocarrier for Drug delivery	Sprague-Dawley rats	Acyclovir loaded in SLPs and Commercial Suspension	SLNs (equivalent to 20 mg/kg of acyclovir) or commercial acyclovir suspension (20 mg/kg of acyclovir) were administered orally. Pharmacological study	Two groups were divided into animals that received SLNs loaded with acyclovir or commercial acyclovir suspension by gavage.	SLNs containing acyclovir exhibited oral bioavailability four times greater than the commercial reference drug.
			**INORGANIC/METALLIC/COMPOSITE NANOPARTICLES**			
**[47]**	AgNPs Virucide: viral inactivation through surface binding	Male or female C57BL/6 mice	Silver nanoparticles surface-modified with EGCG; antiviral action against HSV-1 and HSV-2	HSV-1 was administered intranasally for infection. After 24 and 48 days, the animals received either a NaCl solution containing EGCG-AgNPs or a 0.9% NaCl solution. HSV-2 was inoculated intravaginally. Vaginal washings were performed at 24 and 48 h after infection with EGCG-AgNPs or 0.9% NaCl solution. Biological study	HSV-1 Group of infected mice: EGCG-AgNPs solution or control (saline) HSV-2 Group of infected mice: EGCG-AgNPs solution or control (saline)	NPs were able to inhibit HSV-1 and HSV-2 infection in the models used.
**[50]**	AuNPs Virucide (physical interaction: viral inactivation through surface binding)	CD1 mice	Gold nanoparticles with antiviral action against HSV-1 NPAuG1-S2 NPAuG2-S4 NPAuG3-S8	Mice were injected intravenously in the tail vein with 3.5 mg/Kg of NPAuG3-S8 previously dissolved in PBS and 3 mice were injected only in PBS as control. Pharmacokinetic studies and Bioavailability	Control group treated with PBS and Three mice groups treated with gold NPAuG3-S8	NPAuG3-S8 is able to cross the blood–brain barrier (BBB) and, according to the animal model, does not cause brain damage.
**[51]**	AgNPs or AuNPs/Virucide (immunomodulatory action) (physical interaction: viral inactivation through surface binding)	Female C57BL/6 mice	Silver and gold nanoparticles functionalized with Lactoferrin against HSV-2 Lactoferrin 10 nm LF-AuNPs 10 nm LF-AgNPs 30 nm LF-AgNPs	Vaginal washes were performed 24, 48, and 72 h after infection with 100 µL of saline solution containing 10 µg/mL of LF-Ag/AuNPs or saline solution alone. Biological study	Control group treated with salina solution; other groups treated with Lactoferrin, 10 nm LF-AuNPs, 10 nm LF-AgNPs and 30 nm LF-AgNPs	Lactoferrin-Modified Ag/AuNPs were able to reduce HSV-2 infection; viral titers decreased significantly for all treatments with the nanoparticles in vaginal tissues and spinal cords; Lactoferrin-Modified Ag/AuNPs helped activate the antiviral response.
**[53]**	Tetrapod-shaped ZnO NPs/ Immunomodulation: activation of the immune system against viral antigen Virucide: nanoparticles blocking viral attachment in the cell surface and entry.	Female mice	Tetrapod-shaped ZnO nanoparticles (ZOTEN) against HSV-2	Mice were inoculated with HSV-2 in the presence or absence of ZOTEN Biological study	Groups: uninfected and untreated; UV-inactivated virus alone; ZOTEN and UV-inactivated virus; Alum and UV-inactivated virus; Alum, ZOTEN and UV-inactivated virus	Tetrapod-shaped zinc oxide nanoparticles inhibited HSV-2 by blocking viral attachment and entry. They also induced innate immune cytokines such as IFN-β, IL-6, and TNF-α. The nanoparticles remained on the cell surface. Low cytotoxicity was observed in cell-based assays. Their antiviral action involved both physical blocking and immune stimulation. All infected mice showed lesions 5 days after infection; treatment with ZOTEN showed a significant reduction in lesions and in the viral load in infected mice.
			**FULLERENE/CARBON NANODOTS**			
**[54]**	Fullerene (dnC60)/Virucide (physical interaction): nanomaterial binding to viral surface, preventing the virus from interacting with the cellular receptor, blocking its entry into the cell	Female DBA/2J mice	Water-soluble Fullerene dnC60 and adducts against HSV-1 dnC60 C60-Pip ACV ointment	Mice were infected with HSV-1 and treated with dnC60, C60-Pip, ACV ointment and saline; all tests were applied to the skin once daily for 3 days. Biological study	Groups: dnC60, C60-Pip, ACV ointment and saline	Animals treated with saline showed clinical manifestations of infection, reaching a maximum on days 3 and 6; skin damage was minimal in animals treated with dn60 and ACV; animals treated with C60-Pip showed clinical manifestations and did not recover; a significant decrease in lesions was observed in groups treated with dnC60.
			**LIPOSOMES/NIOSSOMES**			
**[16]**	Liposomes/Nanocarrier for Drug delivery (Gene therapy)	Female BALB/c mice	Liposomes with siRNA for gene therapy for treatment of HSV-1 (LipDOPE-siHSV)	Biodistribution of Lip-siHSV was evaluated in intact mice (fluorescently labeled LipDOPE was injected intraperitoneally) Potential hepatotoxicity of Lip-siHSV was examined following intravenous injection of liposomes. Pharmacokinetic studies and Bioavailability	A control group and a group treated intraperitoneally by injection were used to assess biodistribution; a control group and a group treated intravenously were used to assess hepatotoxicity	No hepatotoxicity was detected after treatment with Lip-siRNAb; biodistribution revealed that LipDOPE accumulated in visceral organs, primarily the liver and spleen (low accumulation was observed in the kidneys, lungs, heart, and central nervous system).
			**NANOEMULSIONS**			
**[60]**	Nanoemulsions/Nanocarrier for Drug delivery (permeation enhancer)	Rats	Nanoemulsions containing acyclovir (ACV-NE hydrogel)	Bioavailability was evaluated in rats after transdermal administration of the nanoemulsions. Pharmacokinetic studies and Bioavailability.	Negative control group treated with plain chitosan hydrogel topically; positive control group treated with reference acyclovir transdermally; group treated with ACV-NE hydrogel transdermally	The total AUC for the ACV-NE hydrogel formulation was significantly higher than that of the commercial acyclovir cream and the raw ACV hydrogel; Cmax was significantly higher for ACV-NE; there was an increase in bioavailability after transdermal administration of ACV-NE.
**[61]**	Nanoemulsions/Nanocarrier for Drug delivery (permeation enhancer)	Male Wistar rats	Garlic oil-acyclovir nanoemulsion (ACV-GO-SNEDDS)	Pharmacokinetics were evaluated after transdermal administration. Pharmacokinetic studies and Bioavailability.	Groups: raw ACV-HPC film, HPC ACV-GO SNEDDs film and ACV cream	ACV-GO SNEDDS transdermal film formulation showed a significant increase in Cmax when compared to raw ACV HPC film and the commercial ACV cream formulation; there was a 2.2-fold increase in relative bioavailability for ACV-GO SNEDDS transdermal film when compared to the commercial form ACV cream, and a 3-fold increase when compared to raw ACV-HPC film; SNEDDS TDDS formulation showed excellent potential for increasing the bioavailability of acyclovir.

Notes: CVM (cervicovaginal mucus); BBB (blood–brain barrier); HPC (hydroxypropylcellulose), NPs (Nanoparticles), PS-PEG (Polystyrene-polyethylene glycol), PCL (Poly-caprolactone), PLGA (Poly-lactic-co-glycolic acid), siRNA (small interfering RNA), EGCG (Epigallocatechin Gallate).

## Data Availability

The original contributions presented in this study are included in the article. Further inquiries can be directed to the corresponding author.
